# Supplementary material for: The “amphi”-brains of amphipods: new insights from the neuroanatomy of Parhyale hawaiensis (Dana, 1853)
Source: Front Zool. 2019 Jul 26;16:30. doi: 10.1186/s12983-019-0330-0 (PMC6660712; doi:10.1186/s12983-019-0330-0)
Supplement: Supplementary file 1 — Table S1. Used protocols for immunohistochemical labelings. (DOCX 17 kb) [file 12983_2019_330_MOESM1_ESM.docx]

**Table S1: Used protocols for immunohistochemical labelings.**

| *vibratome sections (100 µm)* | | *whole-mounts* |
| --- | --- | --- |
| **tubulin + FMRFamide** | **tubulin + histamine** | **synapsin** |
| anaesthetise at 4 °C | | |
| 1. F I X A T I O N | | |
| Bouin’s fixative  until usage at 4 °C  (at least overnight) | 4 % EDAC in PBS + 1 % DMSO  + 5 % glucose  for 30 min. + 24 h at 4 °C | 3 % glyoxal  overnight at 4 °C  (following Richter et al. 2018) |
|  | *post-fixation:* 4 % PFA  for 24 h at 4 °C |  |
| 1. P R E P A R A T I O N | | |
| decapitation | decapitation | dissect brain |
| V I B R A T O M E – S E C T I O N I N G | |  |
| - wash in PBS and transfer into poly-L-lysine - embed in gelatine-ovalbumin - post-fix in 1:20 formaldehyde-PBS at 4 °C | |  |
| 1. P R I M A R Y A N T I S E R A | | |
| wash in PBTx (PBS + 0.5 % Triton X-100 + 1 % bovine serum albumin)  for 2 x 15 min + 60 min at room temperature | | |
| 1:1000 anti-acetylated-tubulin  1:1000 anti-FMRFamide | 1:1000 anti-acetylated-tubulin  1:1000 anti-histamine | 1:1000 anti-SYNORF1 synapsin |
| 1.5 days at room temperature | | 3.5 days at room temperature |
| 1. S E C O N D A R Y A N T I S E R A | | |
| wash in PBTx (0.1 M PBS + 0.5 % Triton X-100 + 1 % BSA)  for 2 x 10 min + 2 x 20 min at room temperature | | |
| 1:500 Cy3 anti-mouse  1:500 Alexa488 anti-rabbit  1:1000 Hoechst 33258 | | 1:500 Cy3 anti-mouse  1:1000 Hoechst 33258 |
| 1.5 days at room temperature | | 2.5 days at room temperature |
| 1. P O S T P R O C E S S I N G | | |
| wash in several changes of 0.1 M PBS (for at least six changes in 3 hours) | | |
| mount in Mowiol 4-88 (Roth 0713.2) | | 1:1 glycerine-PBS for 20 min  4:1 glycerine-PBS for 60 min  mount in 4:1 Dabco-glycerine |
