# Supplementary material for: The “amphi”-brains of amphipods: new insights from the neuroanatomy of Parhyale hawaiensis (Dana, 1853)
Source: Front Zool. 2019 Jul 26;16:30. doi: 10.1186/s12983-019-0330-0 (PMC6660712; doi:10.1186/s12983-019-0330-0)
Supplement: Supplementary file 2 — Table S2. Used reagents for immunohistochemical labelings. (DOCX 17 kb) [file 12983_2019_330_MOESM2_ESM.docx]

**Table S2: Used reagents for immunohistochemical labelings.**

| **Labeling reagent** | **Supplier and specification** |
| --- | --- |
| monoclonal anti-acetylated α-tubulin antibody produced in mouse | Sigma T6793, [1] |
| polyclonal anti-FMRFamide antiserum produced in rabbit | Acris/Immunostar 20091, [1,2] |
| polyclonal anti-histamine antiserum produced in rabbit | Progen 16043, [2–4] |
| monoclonal anti-SYNORF1 synapsin antibody produced in mouse | DSHB 3C11, [1,3] |
| polyclonal Cy3 anti-mouse IgG secondary antibody produced in goat | Jackson Immuno Research 115-165-003, [1] |
| polyclonal Alexa 488 anti-rabbit IgG secondary antibody produced in goat | Invitrogen A11008, [1,3] |
| Hoechst 33258 | Sigma 14530, [1] |

1. Meth R, Wittfoth C, Harzsch S. Brain architecture of the Pacific White Shrimp *Penaeus vannamei* Boone, 1931 (Malacostraca, Dendrobranchiata): correspondence of brain structure and sensory input? Cell Tissue Res. 2017;369:255–71.

2. Harzsch S, Hansson BS. Brain architecture in the terrestrial hermit crab *Coenobita clypeatus* (Anomura, Coenobitidae), a crustacean with a good aerial sense of smell. BMC Neurosci. 2008;9:58.

3. Sombke A, Harzsch S. Immunolocalization of histamine in the optic neuropils of *Scutigera coleoptrata* (Myriapoda: Chilopoda) reveals the basal organization of visual systems in Mandibulata. Neurosci Lett. 2015;594:111–6.

4. Harzsch S, Wildt M, Battelle B, Waloszek D. Immunohistochemical localization of neurotransmitters in the nervous system of larval *Limulus polyphemus* (Chelicerata, Xiphosura): evidence for a conserved protocerebral architecture in Euarthropoda. Arthropod Struct Dev. 2005;34:327–42.
