# Supplementary material for: The “amphi”-brains of amphipods: new insights from the neuroanatomy of Parhyale hawaiensis (Dana, 1853)
Source: Front Zool. 2019 Jul 26;16:30. doi: 10.1186/s12983-019-0330-0 (PMC6660712; doi:10.1186/s12983-019-0330-0)
Supplement: Supplementary file 3 — Table S3. Volume of the brain and reconstructed neuropils of two specimen of P. hawaiensis. (DOCX 14 kb) [file 12983_2019_330_MOESM3_ESM.docx]

**Table S3: Volume of the brain and reconstructed neuropils of two specimen of *P. hawaiensis*.** Measurements are based on histological section series after elastic alignment and montage and, considering this artificial modification, can only serve as an approximate idea. Asterisks indicate neuropils which could not be reconstructed in total due to partial data sets. *Abbreviation:* *hn/tm* hemiellipsoid body/ terminal medulla.

| Reconstructed brain and neuropils | Specimen 1 (male)  volume in µm^3^ | Specimen 2 (female)  volume in µm^3^ |
| --- | --- | --- |
| *Lamina* | 396 842 | 167 689 |
| *Medulla* | 228 437 | 202 578 |
| *Lobula* | 222 496 | 181 789 |
| *Hn/tm-complex* | 1.391.713 | 968 385 |
| *Central body* | 35 122 | 32 814 |
| *Lateral antenna 1 neuropil** | 1 274 361 | 799 561 |
| *Deutocerebral chemosensory lobe* | 430 899 | 317 695 |
| *Antenna 2 neuropil** | 1 840 154 | 752 599 |
| *Total brain** | 19 043 624 | 13 257 517 |
