# Supplementary material for: The “amphi”-brains of amphipods: new insights from the neuroanatomy of Parhyale hawaiensis (Dana, 1853)
Source: Front Zool. 2019 Jul 26;16:30. doi: 10.1186/s12983-019-0330-0 (PMC6660712; doi:10.1186/s12983-019-0330-0)
Supplement: Supplementary file 4 — Interactive content related to Fig. 1c. Three-dimensional reconstruction of a male brain located within the head capsule of P. hawaiensis based on microCT data. (PDF 10294 kb) [file 12983_2019_330_MOESM4_ESM.pdf]

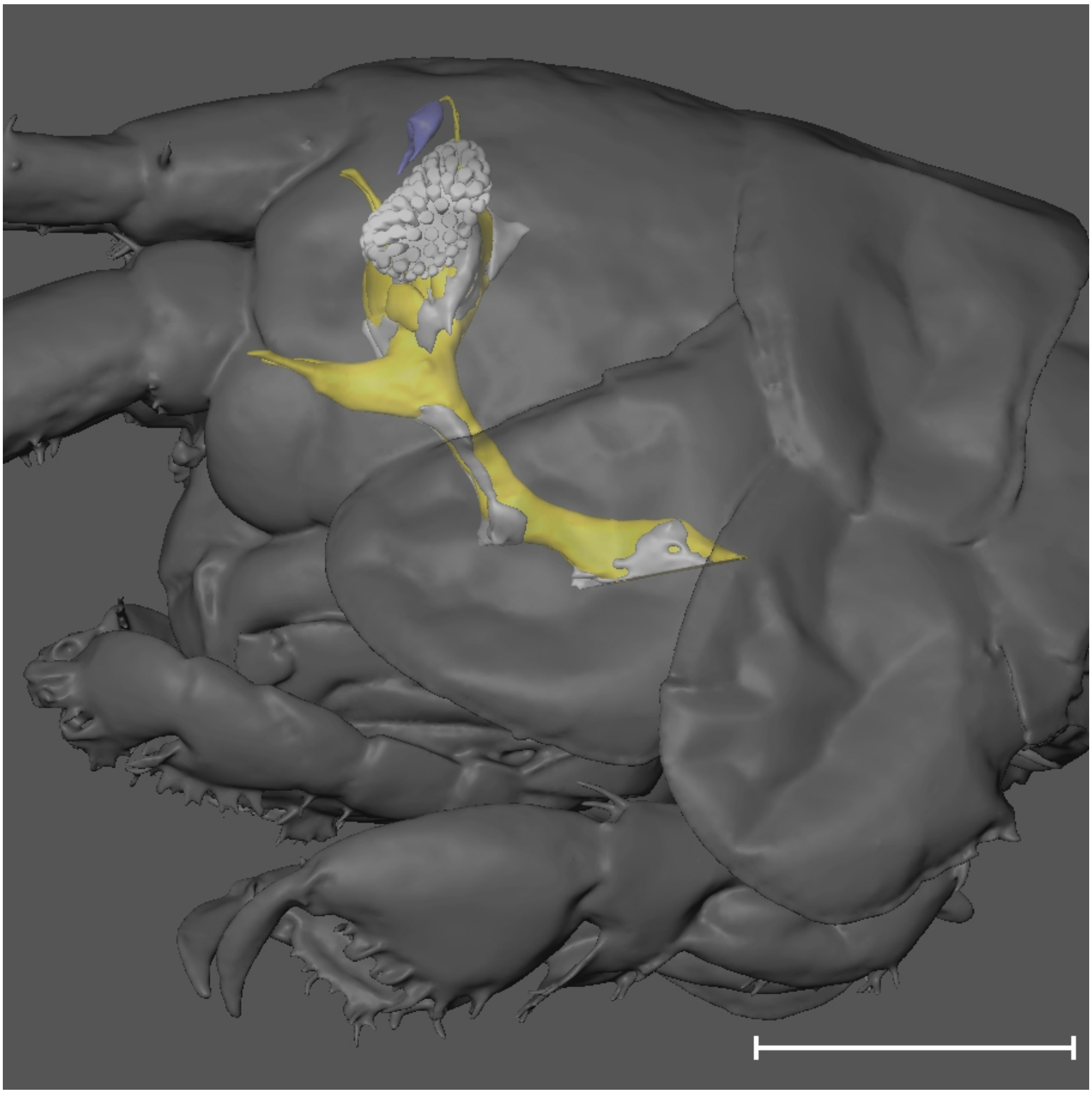

Additional file 1: Interactive content related to figure 1C.Three-dimensional reconstruction of a male brain located within the head capsule of *P. hawaiensis* based on microCT data. The PDF version contains interactive 3D content. To activate, click on the figure in Adobe Reader and by using the computer mouse you can bring the model in any desired position and magnification. Using the model hierarchy, you can in- or exclude all different brain components. For further functionalities see the content menu.
Color code: yellow: brain neuropil, gray: brain cortex, white: optic system, blue: organ of Bellonci. Scale bar: 500µm
