# Supplementary material for: The “amphi”-brains of amphipods: new insights from the neuroanatomy of Parhyale hawaiensis (Dana, 1853)
Source: Front Zool. 2019 Jul 26;16:30. doi: 10.1186/s12983-019-0330-0 (PMC6660712; doi:10.1186/s12983-019-0330-0)
Supplement: Supplementary file 6 — Interactive content related to Fig. 7. Three-dimensional reconstruction of a male brain of P. hawaiensis based on histological sections. (PDF 6177 kb) [file 12983_2019_330_MOESM6_ESM.pdf]

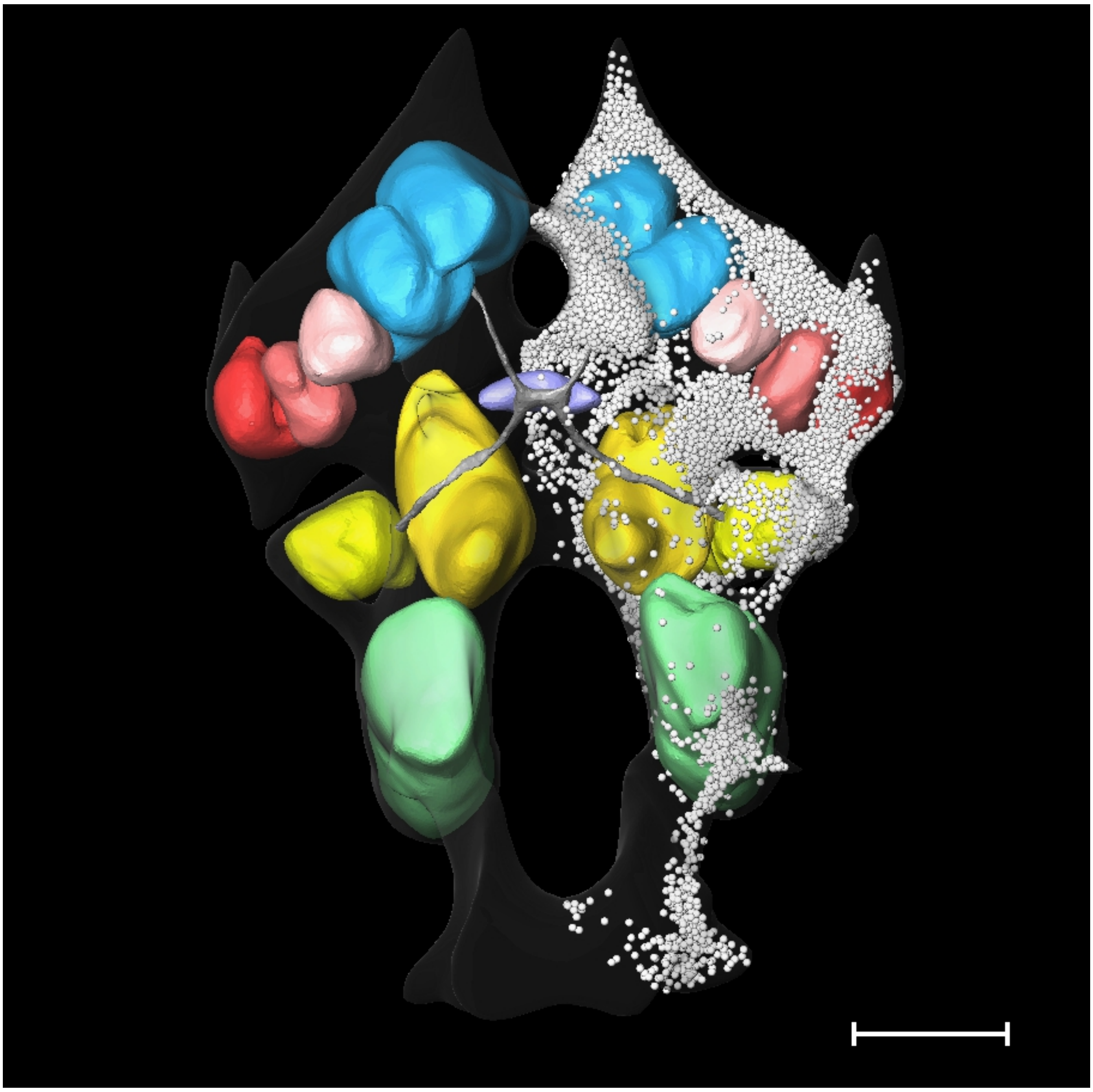

Additional file 3: Interactive content related to figure 7. Three-dimensional reconstruction of a male brain of *P. hawaiensis* based on histological sections. The PDF version contains interactive 3D content. To activate, click on the figure in Adobe Reader and by using the computer mouse you can bring the model in any desired position and magnification. Using the model hierarchy, you can in- or exclude all different brain components. For further functionalities see the content menu.

Abbreviations: ann antenna 2 neuropil, cb central body, dcl deutocerebral chemosensory lobe, hn/tm hemiellipsoid body/ terminal medulla complex, la lamina, lan lateral antenna 1 neuropil, lo lobula, me medulla, pnt projection neuron tract. The nuclei of the brain (somata) only shown in the right hemisphere. Scale bar: 100µm
